# Long-Term Outcomes and Determinants of New-Onset Mental Health Conditions After Trauma

**DOI:** 10.1001/jamanetworkopen.2025.0349

**Published:** 2025-03-10

**Authors:** Lai Kin Yaw, Maxine Burrell, Kwok Ming Ho

**Affiliations:** 1 Department of Intensive Care, Fiona Stanley Hospital, Murdoch, Western Australia, Australia; 2 Department of Emergency Medicine, Royal Perth Hospital, Perth, Western Australia, Australia; 3 Retired, State Trauma Unit, Royal Perth Hospital, Perth, Western Australia, Australia; 4 Department of Anaesthesia and Intensive Care, The Chinese University of Hong Kong, Hong Kong SAR, China; 5 The Prince of Wales Hospital, Hong Kong SAR, China; 6 The Peter Hung Pain Research Institute, the Chinese University of Hong Kong, Hong Kong SAR, China

## Abstract

**Importance:**

Evidence suggests that trauma-related mortality and morbidities may follow a multiphasic pattern, with outcomes extending beyond hospital discharge.

**Objectives:**

To determine the incidence of having new mental health conditions after the first (or index) trauma admission and their association with long-term health outcomes.

**Design, Setting, and Participants:**

This population-based, linked-data cohort study was conducted between January 1994 and September 2020, with data analyzed in April 2024. Participants were adult patients with trauma admitted to 1 of the 5 adult trauma hospitals in Western Australia. All patients with major trauma with an Injury Severity Score (ISS) greater than 15 were included. For each patient with major trauma, 2 patients with trauma with a lower ISS (<16) were randomly selected.

**Exposure:**

A new mental health condition recorded in either subsequent public or private hospitalizations after trauma admission.

**Main Outcomes and Measures:**

The primary outcomes were the associations between new mental health conditions after trauma and subsequent risks of trauma readmission, suicide, and all-cause mortality, as determined by Cox proportional hazards regression. Logistic regression was used to determine which factors were associated with developing a new mental health condition after trauma.

**Results:**

Of 29 191 patients (median [IQR] age, 42 [27-65] years; 19 383 male [66.4%]; median [IQR] ISS, 9 [5-16]; 9405 with ISS >15 and 19 786 with ISS <16) considered, 2233 (7.6%) had a mental health condition before their trauma admissions. The median (IQR) follow-up time after the index trauma admission was 99.8 (61.2-148.5) months. Of 26 958 patients without a prior mental health condition, 3299 (11.3%) developed a mental health condition subsequently, including drug dependence (2391 patients [8.2%], with 419 patients [1.4%] experiencing opioid dependence) and neurotic disorders (1574 patients [5.4%]), including posttraumatic stress disorder. Developing a new mental health condition after trauma was associated with subsequent trauma readmissions (adjusted hazard ratio [aHR], 1.30; 95% CI, 1.23-1.37; *P* < .001), suicides (aHR, 3.14; 95% CI, 2.00-4.91; *P* < .001), and all-cause mortality (aHR, 1.24; 95% CI, 1.12-1.38; *P* < .001). Younger age, unemployment, being single or divorced (vs married), Indigenous ethnicity, and a lower socioeconomic status were all associated with developing a new mental health condition after the first trauma admission.

**Conclusions and Relevance:**

This cohort study of 29 191 patients with trauma found that mental health conditions after trauma were common and associated with an increased risk of adverse long-term outcomes, indicating that mental health follow-up of patients with trauma, particularly those from vulnerable subgroups, may be warranted.

## Key Points

**Question:**

Compared with before trauma admission, are patients with trauma at risk of developing a new mental health condition, and does this affect their long-term health outcomes?

**Findings:**

In this cohort study of 29 191 patients with trauma, 3299 (11.3%) developed a new mental health condition subsequently, which was associated with long-term trauma readmissions, suicides, and all-cause mortality. Younger age, unemployment, marital status, Indigenous ethnicity, and lower socioeconomic status were associated with developing a new mental health condition after trauma.

**Meaning:**

These findings indicate that mental health follow-up of patients with trauma, particularly in vulnerable subgroups, may be warranted.

## Teaser

This cohort study examines the incidence of new mental health condition diagnoses after a first (or index) trauma admission and the impact on long-term health outcomes.

## Introduction

Injury accounts for substantial morbidity and mortality worldwide. The cost of care for traumatic injuries is staggering.^1,2^ The development of trauma systems and designated trauma centers has been proven to save lives of patients who would have died without advanced trauma care.^3^ Nonetheless, recent evidence suggests that trauma mortality and morbidities may follow a multiphasic pattern, with increasing number of survivors experiencing long-term morbidities after hospital discharge.

Trauma most commonly affects young persons with few preexisting diseases; yet, the results from previous studies^4,5,6,7,8^ have shown that severely injured patients have a higher cumulative mortality in the years following the initial trauma admission. Many of the severely injured patients sustain trauma involving several body systems. They are at risk of developing long-term physical, mental, and psychological disabilities after their trauma admission. Previous studies^9,10,11,12^ have described adverse consequences, including the inability to return to previous levels of activity or employment and delayed mortality due to long-term complications of trauma, a second episode of major trauma, or suicides.

The mental health issues following trauma are not limited to posttraumatic stress disorder (PTSD).^13^ Other mental health conditions, such as suicidality, depression, and substance abuse, have also been reported as sequelae of trauma.^14^ The Victoria State Trauma Registry in Australia reported on 2757 adult patients with trauma and found that 50% of the survivors experienced ongoing pain or discomfort, and 41% had symptoms of anxiety or depression 3 years after their injuries.^15^ Although long-term health sequelae of trauma are common and substantial, most trauma hospitals and registries currently do not track patients’ trajectories after trauma hospitalizations, and longitudinal studies of the long-term health outcomes of seriously injured people are sparse. A 2018 Canadian study^16^ in Ontario with 19 338 patients examined mental health outcomes within 5 years following trauma and found that the survivors of major trauma were at a heightened risk of developing mental health conditions or death by suicide in the years after their injury. Maximizing long-term outcomes, based on data from longitudinal studies, including identifying which groups may benefit the most from intervention after trauma center discharge, is considered the next important challenge that trauma centers must face.^17^

Currently, the long-term incidence and determinants of developing new mental health conditions after trauma beyond 5 years of follow-up, including PTSD, neurotic disorders, and suicides, remain uncertain. Furthermore, whether developing new mental health conditions after trauma admission contributes to trauma readmissions and long-term mortality has not been investigated. We hypothesized that among those without a mental health condition before trauma admission, developing a new-onset mental health condition is common, and this could be associated with an increased risk of adverse long-term outcomes. In this study, we aimed to report the incidence and determinants of developing a mental health condition after trauma and its potential long-term association with risk of suicides, trauma readmissions, and all-cause mortality.

## Methods

### Study Design

This was a retrospective, population-based, linked-data cohort study of adult patients with trauma in the state of Western Australia. All patients aged 17 years and older with traumatic injuries with an Injury Severity Score (ISS) greater than 15 recorded in the Western Australia’s State Trauma Registry from January 1994 until September 2020 were included; in addition, for each patient with major trauma (ISS >15) included, 2 patients with trauma with a lower severity of injury (ISS <16) were randomly selected for this cohort study. This study was approved by the Department of Health Ethics Committee. Owing to the large number of patients included and the release of only deidentified data to the researchers, a waiver of consent was permitted by the Ethics Committee as per Section 2.3.10 of the Australian National Statement regarding medical research ethics. We adhered to the Strengthening the Reporting of Observational Studies in Epidemiology (STROBE) reporting guidelines in reporting our results.

### Source of Data

The Western Australia’s State Trauma Registry is a comprehensive dataset consisting of in-hospital events, from the time of trauma to discharge from the hospital and rehabilitation services. Until 2011, Trauma Registry data were collected individually on separate, but identical, Microsoft Access databases by the individual trauma hospitals. In 2011, all trauma registry data were combined into a single web-based database, the Western Australia State Trauma Registry. The criteria for inclusion into the registry are as follows: all patients with major and minor trauma who present to a hospital for treatment within 7 days of their date of trauma and who were hospitalized for greater than 24 hours at that hospital, unless trauma-related deaths occur within 24 hours of admission. Our cohort included patients who were aged 17 years and older and admitted to 1 or more of the following trauma hospitals in Western Australia: Royal Perth Hospital (1994 to 2020), Fremantle Hospital (2011 to 2020), Joondalup Hospital (2011 to 2020), Sir Charles Gairdner Hospital (2011 to 2020), and Fiona Stanley Hospital (2015 to 2020).

### Data Linkage

To ascertain a comprehensive picture of the trajectory of each individual patient with trauma, we linked the Trauma Registry data with the Department of Health Hospital Morbidity Data Set, Emergency Department Data Set, Mental Health Dataset, and Death Registry, including a 5-year look-back period before the first (or earliest arrival date) trauma admission. The 5-year look-back period aimed to ascertain whether subsequent clinical sequelae were related to preexisting conditions before the first (or index) trauma admission. The Health Hospital Morbidity Data Set contains a vast amount of health data including hospitalization data, such as length of stay, primary admission diagnosis, other codiagnoses, and procedures performed in all public as well as private hospital admissions, allowing us to determine comorbidities (eg, Charlson Comorbidity Index and mental health conditions) both before and after the index trauma admissions using *International Statistical Classification of Diseases, Tenth Revision, Clinical Modification* codes. The Death Registry data were used to ascertain the causes of death, including whether it was suicide related. In this study, the censor date for mortality was December 11, 2020. In addition, we also obtained the socioeconomic status index at the local government areas on the basis of each patient’s residential address at the time of index trauma admission.^18^

### Statistical Analysis

Data were analyzed in April 2024 using SPSS for Windows version 29.0.2.0 (IBM), MedCalc for Windows version 12.5 (MedCalc Software, Ltd), or S-PLUS version 8.0 (Insightful Corp). Cox proportional hazards regression was used to determine factors associated with suicide after trauma, all-cause mortality, and trauma readmission. Logistic regression was used to determine factors associated with developing a new mental health condition after trauma. In choosing covariates for adjustment in the Cox and logistic regression analyses, only biologically plausible factors for these outcomes were included, and factors with an associated *P* > .25 were removed to generate the final parsimony model. In determining factors associated with a new-onset mental health condition after trauma, a subgroup analysis was also conducted among those with smoking and alcohol use data. All tests were 2-tailed, and *P* < .05 was considered significant without adjusting for multiple statistical testing.

## Results

### Characteristics of the Cohort

**Figure 1.**
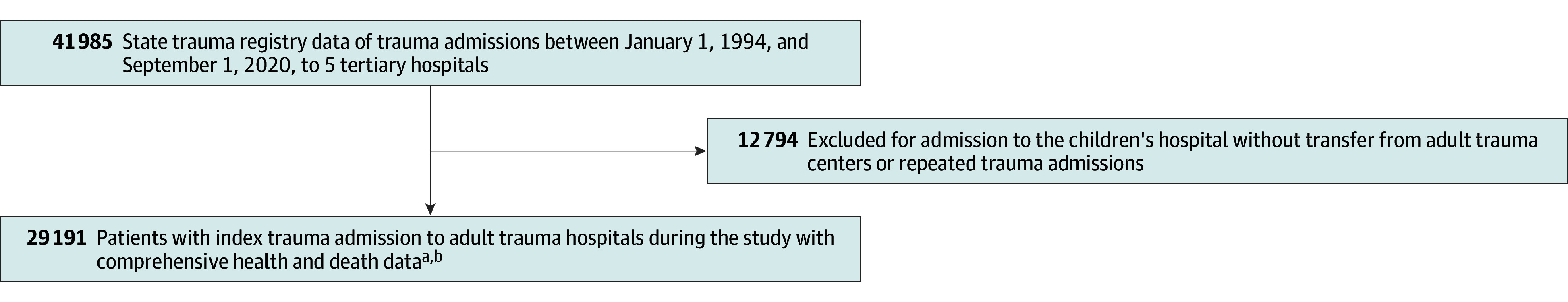
Patient Enrollment Flowchart ^a^Linkage with the statewide Department of Health Hospital Morbidity Data Set and death registry was performed for 29 191 patients with health data from 426 981 public or private hospitalization up to 5 years before and after their index trauma admissions until the censor date (December 11, 2020). ^b^There were 65 555 public or private hospitalizations before and 198 395 hospitalizations after study patients' index trauma admissions; 3868 patients (13.2%) died on or before the censor date.

Of the 29 191 adult patients with trauma included in the study (Figure 1), a total of 65 555 public or private hospitalizations before and 198 395 hospitalizations after the first (or index) trauma admissions were analyzed. Male patients were overrepresented (19 383 men [66.4%]). The median (IQR) age was 42 (27-65) years, the median (IQR) ISS was 9 (5-16) (9405 patients had ISS >15 and 19 786 had ISS <16), and the median (IQR) follow-up time for the cohort was 99.8 (61.2-148.5) months; 3868 patients (13.2%) died on or before the censor date. Of the 29 191 patients, 9010 (30.9%) had subsequent trauma admissions during the follow-up period after their first trauma admissions. The characteristics of the cohort are described in Table 1.

**Table 1.** Characteristics of the Cohort

Characteristic	Patients, No. (%)		
	Total (N = 29 191)	Patients without mental health condition before index trauma admission (n = 26 958)	
		Developed new mental health condition (n = 3299)	Did not develop new mental health condition (n = 23 659)
Age, median (IQR), y	42 (27-65)	36 (25-53)	43 (27-67)
Sex			
Female	9808 (33.6)	1038 (31.5)	7888 (33.3)
Male	19 383 (66.4)	2261 (68.5)	15 771 (66.7)
Ethnicity			
Indigenous	2303 (7.9)	766 (23.2)	1142 (4.8)
Other	26 888 (92.1)	2533 (76.8)	22 517 (95.2)
Marital status at the time of injury			
Never married	12 227 (41.9)	1736 (52.6)	9454 (40.0)
Married or de facto	11 821 (40.5)	1025 (31.1)	10 111 (42.7)
Widowed	2590 (8.9)	185 (5.6)	2223 (9.4)
Divorced	1164 (4.0)	128 (3.9)	862 (3.6)
Separated	699 (2.4)	121 (3.7)	459 (1.9)
Unknown	690 (2.4)	104 (3.2)	550 (2.3)
Injury Severity Score, median (IQR)	9 (5-16)	9 (5-16)	9 (5-16)
Unemployed at the time of first trauma admission	2351 (8.1)	592 (17.9)	1319 (5.6)
Socioeconomic score according to local government areas of the patient’s address, median (IQR)^a^	1019 (987-1033)	1009 (985-1028)	1021 (994-1033)
Smoker^b^	1336 (13.6)	242 (11.4)	934 (3.9)
Alcohol user^b^	1714 (17.5)	284 (13.4)	1232 (5.2)
Blunt injury	26 644 (91.3)	2990 (90.6)	21 626 (91.4)
Principal injured site			
Head	6954 (23.8)	928 (28.1)	5371 (22.7)
Neck (including cervical spine)	1471 (5.0)	154 (4.7)	1218 (5.1)
Thorax (including thoracic spine)	2214 (7.6)	259 (7.9)	1786 (7.5)
Abdomen and pelvis (including lumbar spine)	2655 (9.1)	276 (8.4)	2196 (9.3)
Upper limbs (including shoulders)	8327 (28.5)	922 (27.9)	6830 (28.9)
Lower limbs	6530 (22.4)	621 (18.8)	5444 (23.0)
Burns	861 (2.9)	107 (3.2)	681 (2.9)
Foreign body penetrating natural orifices	120 (0.4)	21 (0.6)	89 (0.4)
Nonspecified or others	59 (0.2)	8 (0.2)	36 (0.2)
Charlson Comorbidity Index before trauma admission, median (IQR)^c^	2 (1-2)	0 (0-0)	0 (0-0)
Comorbidity before index trauma admission			
Ischemic heart disease	674 (2.3)	49 (1.5)	543 (2.3)
Congestive heart failure	788 (2.7)	48 (1.5)	635 (2.7)
Cerebrovascular event	143 (0.5)	9 (0.3)	102 (0.4)
Chronic pulmonary disease	826 (2.8)	84 (2.5)	543 (2.3)
Connective tissue disease	23 (0.1)	0	20 (0.1)
Diabetes with complications	829 (2.8)	67 (2.0)	653 (2.8)
Diabetes without complications	732 (2.5)	78 (2.4)	521 (2.2)
Hematologic cancer	38 (0.1)	3 (0.1)	35 (0.1)
Cancers	1077 (3.7)	61 (1.8)	913 (3.9)
Metastatic cancers	178 (0.6)	10 (0.3)	146 (0.6)
Length of hospital stay, median (IQR), d	3 (1-7)	2 (1-6)	3 (1-7)
30-d Mortality	611 (2.1)	0	553 (2.3)
1-y Mortality	1387 (4.8)	26 (0.8)	1202 (5.1)
Subsequent trauma admissions	9010 (30.9)	1821 (55.2)	6307 (26.7)
No. of subsequent trauma readmissions (among those with trauma readmissions), median (IQR)	1 (1-2)	1 (0-1)	0 (0-1)
Mortality (on or before censor date, December 11, 2020)	3868 (13.2)	400 (12.1)	3007 (12.7)
Follow-up time until censor date or death after index trauma admission, median (IQR), mo	99.8 (61.2-148.5)	124.1 (83.5-170.8)	96.7 (59.2-145.4)

^a^ A higher score indicates more advantaged according to socioeconomic factors, standardized to a distribution where the mean equals 1000 and the SD is 100.^18^

^b^ Only data from 9820 patients were available.

^c^ Only 3713 patients had 1 or more of the Charlson Comorbidity Index conditions.

### Differences in Mental Health Diagnoses Before and After Index Trauma Admission

In total, 2233 patients (7.6%) had a mental health condition before their index trauma admission. Among the 26 958 patients (92.4%) who did not have a prior mental health condition, 3299 (11.3%) developed a mental health condition after their index trauma admission.

There was a significant increase in number of hospitalizations with a mental health diagnosis after the first trauma admission (557 hospitalizations per 1000 patients; 95% CI, 499-614 hospitalizations per 1000 patients; *P* = .001), with most substantial increases for hospitalizations for drug dependence and neurotic disorders (Table 2). Among 26 958 patients without any mental health conditions before the first trauma admission, 1574 (5.4%) developed a neurotic disorder, including PTSD, and 2391 (8.2%) developed drug dependence diagnosis after trauma. Specifically, 130 patients (0.5%) were opioid-dependent before the index trauma admission but another 419 patients (1.4%) became opioid dependent after their index trauma admissions. One hundred sixty-two patients (0.6%) had anxiety disorders before the index trauma admission; after the index trauma admissions, 896 patients (3.1%) who did not have anxiety disorders before developed anxiety disorders.

**Table 2.** Differences in Number of Patients With Various Mental Health Diagnoses and Hospitalizations Before and After Their First (Index) Trauma Admission

Mental health diagnosis	Patients, No. (%) (N = 29 191)			Difference in No. of hospitalizations before and after trauma/1000 patients, mean (95% CI)	*P* value^b^
	Hospitalized with mental health condition before trauma	Hospitalized with mental health condition after trauma^a^	Diagnosed with mental health condition only after trauma		
Neurotic disorders	1023 (3.5)	1965 (6.7)	1574 (5.4)	81 (64 to 98)	.001
Posttraumatic stress disorder	97 (0.3)	269 (0.9)	237 (0.8)	16 (11 to 21)	.001
Drug dependence	1467 (5.0)	3311 (11.3)	2391 (8.2)	399 (357 to 442)	.001
Mood disorders	312 (1.1)	502 (1.7)	393 (1.3)	18 (10 to 26)	.001
Deliberate self-harm	21 (0.2)	164 (0.6)	163 (0.6)^c^	6 (5 to 7)^d^	.001
Behavioral syndromes	52 (0.2)	97 (0.3)	85 (0.3)	1 (−3 to 5)	.61
Personality disorders	206 (0.7)	449 (1.5)	390 (1.3)	23 (17 to 28)	.001
Schizophrenia	221 (0.8)	475 (1.6)	345 (1.2%)	33 (25 to 41)	.001
Any of these mental diagnoses	2233 (7.6)	4652 (15.9)	3299 (11.3)^e^	557 (499 to 614)	.001

^a^ Including patients who had mental health conditions before trauma; note that some of these patients did not have subsequent hospitalizations with the same mental diagnoses after trauma.

^b^ Generated by paired *t* test.

^c^ Patients who died from suicide subsequent to trauma index admission were included.

^d^ The admissions due to suicide after index trauma admission were not included.

^e^ Did not include posttraumatic stress disorder (as it was one of the neurotic disorders) and also patients who completed suicide.

### Associations Between Having a New Mental Health Condition After Trauma and Subsequent Risks of Suicide, Trauma Readmission, and All-Cause Mortality

**Figure 2.**
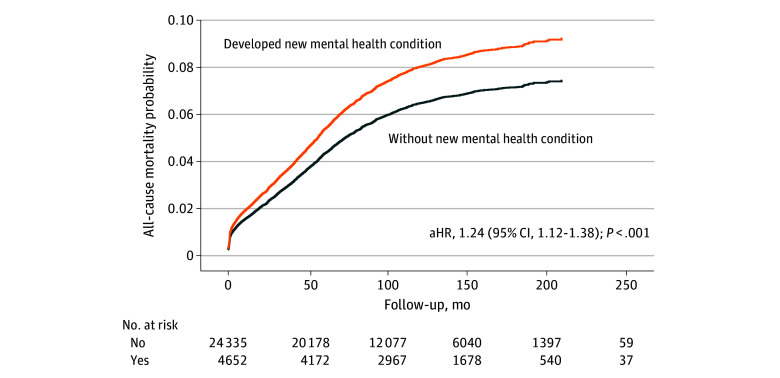
Multivariable Cox Regression Showing the Difference in Risk of All-Cause Mortality Between Those Who Did vs Did Not Develop a Mental Health Condition Following Trauma Models were adjusted for age, Injury Severity Score, sex, employment status, socioeconomic score, Charlson Comorbidity Index, and presence or absence of a mental health condition before index trauma admission. aHR indicates adjusted hazard ratio.

Having a new mental health condition after trauma was associated with subsequent trauma readmissions (adjusted hazard ratio [aHR] 1.30; 95% CI, 1.23-1.37; *P* < .001), suicide by hanging or drug overdose (aHR, 3.14; 95% CI, 2.00-4.91; *P* < .001), and all-cause mortality (aHR, 1.24; 95% CI, 1.12-1.38; *P* < .001) (Figure 2) after adjusting for age, sex, and whether the patients had a mental health condition before the index trauma admission (Table 3). See eFigure 1 and eFigure 2 in Supplement 1 for more details.

**Table 3.** Multivariable Cox Models Showing Associations Between the Presence of a Mental Health Condition Before Trauma, a Newly Developed Mental Health Condition, and Risk of Suicide by Hanging or Drug Overdoses, Trauma Readmission, and All-Cause Mortality

Outcome and variables	β coefficient	SE	Wald statistic	aHR (95% CI)	*P* value
Suicide by hanging or drug overdoses (n = 115)^a^					
Developed a mental health condition after trauma (before completing suicide) among those without a mental health condition before trauma	1.144	0.228	25.19	3.14 (2.01-4.91)	<.001
Presence of a mental health condition before trauma admission	1.823	0.229	63.49	6.19 (3.95-9.69)	<.001
Age (per year increment)	−0.010	0.0053	3.62	0.99 (0.98-1.00)	.06
Female sex	−0.662	0.246	7.24	0.52 (0.32-0.84)	.007
ISS (per score increment)	0.078	0.005	249.37	1.08 (1.07-1.09)	<.001
Repeated trauma admissions (n = 9010)^b^					
Developed a mental health condition after trauma among those without a mental health condition before trauma	0.262	0.028	85.13	1.30 (1.23-1.37)	<.001
Presence of a mental health condition before trauma admission	0.279	0.037	55.77	1.32 (1.23-1.42)	<.001
Age (per year increment)	0.004	0.001	29.15	1.01 (1.00-1.01)	<.001
Female sex	−0.081	0.0260	9.66	0.92 (0.88-0.97)	.002
ISS (per score increment)	0.009	0.002	19.94	1.01 (1.01-1.01)	<.001
Local government areas socioeconomic score according to patient’s residential address (per score increment)^c^	−0.001	0.001	2.65	0.99 (0.99-1.00)	.10
Unemployed (vs employed) at the time of trauma index admission	0.278	0.039	51.60	1.32 (1.22-1.43)	<.001
All-cause mortality (n = 3868)^d^					
Developed a mental health condition after trauma among those without a mental health condition before trauma	0.216	0.055	15.58	1.24 (1.12-1.38)	<.001
Presence of a mental health condition before trauma admission	0.513	0.052	97.77	1.70 (1.51-1.85)	<.001
Age (per year increment)	0.051	0.001	1398.56	1.05 (1.05-1.06)	<.001
Female sex	−0.189	0.035	28.62	0.83 (0.77-0.89)	<.001
ISS (per score increment)	0.051	0.002	883.18	1.05 (1.05-1.06)	<.001
Charlson Comorbidity Index before trauma (per score increment)	0.141	0.009	258.25	1.15 (1.13-1.17)	<.001
Local government areas socioeconomic status score according to patient’s residential address (per score increment)^c^	0.002	0.001	25.45	1.00 (1.00-1.00)	<.001
Unemployed (vs employed) at the time of trauma index admission	1.041	0.104	100.01	2.83 (2.31-3.47)	<.001

Abbreviations: aHR, adjusted hazard ratio; ISS, Injury Severity Score.

^a^ The Harrell C-index of the model was 0.812 (95% CI, 0.768-0.856). Employment status at the time of trauma index admission (*P* > .25) and patient residential address’s local government areas socioeconomic status score (*P* > .25) were not significantly associated with increased risk of suicide, and adding them to the model did not change the positive associations between the 2 mental health variables and risk of suicide.

^b^ The Harrell C-index of the model was 0.606 (95% CI, 0.599-0.613).

^c^ A higher score indicates more advantaged based on socioeconomic factors, standardized to a distribution where the mean equals 1000 and the SD is 100.^18^ Age was significantly correlated with socioeconomic score in the study cohort (Pearson coefficient = 0.15; *P* < .001).

^d^ The Harrell C-index of the model was 0.853 (95% CI, 0.847-0.859).

### Factors Associated With Having a New Mental Health Condition After Trauma Among Those Without Any Mental Health Diagnosis Before Trauma

Among 26 958 patients without any mental health condition before their index trauma admissions, young age, unemployment, single or divorced or separated status (vs married), Indigenous ethnicity, lower socioeconomic status, and traumatic brain injury were all associated with developing a new mental health condition after their first trauma admissions (eTable in Supplement 1). Subgroup analysis on those who had alcohol and smoking use data yielded similar results, and both smoking and alcohol use were significantly associated with an increased risk of developing a new mental health condition after index trauma admissions.

## Discussion

It is recognized that immediate medical care following trauma represents only a fractional aspect of what most injured patients may need.^17^ Understanding the long-term outcomes and why some individuals are at risk of having worse long-term outcomes after trauma are essential to optimize patients’ recovery from the injuries and ability to reintegrate into society. To our knowledge, this cohort study is the largest and longest longitudinal study describing mental health outcomes in severely injured patients to date.

This study confirmed that new mental health conditions after trauma were common and associated with an increased risk of adverse long-term health outcomes, including a higher risk of trauma readmission, suicide, and all-cause mortality. These findings have some clinical relevance and require further discussion.

First, the patient characteristics in our cohort, including relatively young age (median age, 42 years) and male patients being overrepresented, were similar to previous studies.^15,16,17^ Similar to the Ontario study by Evans et al,^16^ patients with mental health conditions before index trauma admission accounted for approximately 7% of the study cohort. More importantly, the trajectories of our patients followed a multiphasic pattern. Approximately 13% of all patients discharged alive after trauma had died subsequently, and nearly one-third of our patients had subsequent trauma readmissions after a median follow-up period of 99.8 months.

Second, our results showed that a significant number of patients with trauma developed a new mental health condition after their first trauma admission. Aside from PTSD, there was a high incidence of drug dependence, other neurotic disorders, and mood disorders. Patients with trauma are at risk of having chronic pain, functional limitations, and reduced physical health, and these issues could contribute to the development of new mental health conditions.^13^ A previous study by Heimke et al^19^ found that mental illness and recreational drug use were factors associated with trauma recidivism. It is, therefore, not surprising that the patients who developed new mental health conditions following trauma have higher risk of trauma readmissions, drug overdoses, and suicides. Our study showed that patients who developed a new mental health condition after trauma had a 3-fold increase in the risk of dying by suicide by either hanging or drug overdoses. Because having new mental health conditions after trauma was associated with an increased risk of repeated trauma hospitalizations and suicides, a higher risk for all-cause mortality was, therefore, also not surprising.

Third, because trauma outcomes follow a multiphasic trajectory beyond the immediate hospitalization for trauma,^17^ identification of which groups may benefit the most from intervention after trauma center discharge is of pivotal importance. Our study showed that some patient factors are associated with their risk of developing a new mental health condition after trauma among those without any mental health condition before the index trauma admissions. Younger age, unemployment, being single or divorced and/or separated (vs married), Indigenous ethnicity, lower socioeconomic status, and traumatic brain injury were all associated with the development of a new mental health condition after their trauma admission. It is increasingly clear that mental health conditions are related to multiple upstream causes, including socioeconomic factors.^20^ On the other hand, occurrence of trauma and trauma outcomes are also related to socioeconomic factors.^21,22^ As such, mental health conditions, trauma, and socioeconomic factors are likely all interrelated like a triad, with a potential to exacerbate the adverse effects of each domain on patient outcomes including long-term mortality.

### Limitations

We need to acknowledge the limitations of this study. The Western Australian State Trauma Registry captures the majority of trauma admissions and virtually all major trauma admissions to the public health care system in Western Australia. However, patients who remained in regional hospitals due to minor injuries that did not require specialized care were not included in this study. We might, therefore, underestimate the importance of rurality as a risk factor for developing mental health conditions after trauma. By capturing only injuries requiring inpatient admissions (excluding patients with minor injuries treated in an emergency department and discharged home, which can constitute a meaningful fraction of patients seen at many trauma hospitals), and only capturing mental health conditions diagnosed at a subsequent hospitalization (excluding patients who have mental health conditions diagnosed by an outpatient practitioner, which may be a relatively large cohort), both of these limitations may lead to underestimation of the incidence and impact of these postinjury mental health conditions. This makes the work presented here all the more important, as the problem described is likely even larger than reported. Because data regarding prescriptive drug use, active substance abuse, and recreational drug use were not well captured by this database, we were unable to determine whether substance use beyond smoking and alcohol may be a risk factor in developing mental health conditions following trauma, nor its association with the risk of suicide and all-cause mortality.

## Conclusions

In this cohort study of 29 191 patients with trauma, we found that mental health conditions after trauma were common and associated with an increased risk of adverse long-term outcomes. Trauma outcomes clearly followed a multiphasic pattern beyond immediate trauma care. Identifying groups who may benefit the most from interventions after trauma center discharge is important. Given the burden of new-onset mental conditions after trauma and their substantive long-term impact on the risk of trauma readmission, suicide, and all-cause mortality, there is potential to improve long-term outcomes for patients with trauma if appropriate mental health care resources are in place to offer follow-up and targeted interventions, including smoking cessation, alcohol use counseling, and treatment of opioid dependence, particularly for vulnerable subgroups of patients with trauma.

## Notes

Supplement 1. **eFigure 1.** Univariable Cox regression showing the difference in risk of suicide by hanging or drug overdoses between those who developed a mental health condition and those who did not after their index trauma admission **eFigure 2.** Multivariable Cox regression showing the difference in risk of suicide by hanging or drug overdoses between those who developed a mental health condition and those without did not after their index trauma admission, after adjusted for age, Injury Severity Score (ISS), sex, and presence or absence of a mental health condition before the index trauma admission **eTable.** Predictors of developing a new mental health diagnosis after trauma among those who did not have any mental health conditions before their index trauma admissions (N=26,958)

Supplement 2.
Data Sharing Statement
