# Supplementary material for: Long-Term Outcomes and Determinants of New-Onset Mental Health Conditions After Trauma
Source: JAMA Netw Open. 2025 Mar 10;8(3):e250349. doi: 10.1001/jamanetworkopen.2025.0349 (PMC11894494; doi:10.1001/jamanetworkopen.2025.0349)
Supplement: Supplement 1. — eFigure 1. Univariable Cox regression showing the difference in risk of suicide by hanging or drug overdoses between those who developed a mental health condition and those who did not after their index trauma admission eFigure 2. Multivariable Cox regression showing the difference in risk of suicide by hanging or drug overdoses between those who developed a mental health condition and those without did not after their index trauma admission, after adjusted for age, Injury Severity Score (ISS), sex, and presence or absence of a mental health condition before the index trauma admission eTable. Predictors of developing a new mental health diagnosis after trauma among those who did not have any mental health conditions before their index trauma admissions (N=26,958) [file jamanetwopen-e250349-s001.pdf]

Supplemental Online Content

Yaw LK, Burrell M, Ho KM. Long-term outcomes and determinants of new-onset mental health conditions after trauma. *JAMA Netw Open*. 2025;8(3):e250349. doi:10.1001/jamanetworkopen.2025.0349

eFigure 1. Univariable Cox regression showing the difference in risk of suicide by hanging or drug overdoses between those who developed a mental health condition and those who did not after their index trauma admission
eFigure 2. Multivariable Cox regression showing the difference in risk of suicide by hanging or drug overdoses between those who developed a mental health condition and those without did not after their index trauma admission, after adjusted for age, Injury Severity Score (ISS), sex, and presence or absence of a mental health condition before the index trauma admission
eTable. Predictors of developing a new mental health diagnosis after trauma among those who did not have any mental health conditions before their index trauma admissions (N=26,958)

This supplemental material has been provided by the authors to give readers additional information about their work.

.
© 2025 Yaw LK et al. *JAMA Network Open*.

eFigure 1. Univariable Cox regression showing the difference in risk of suicide by hanging or drug overdoses between those who developed a mental health condition and those who did not after their index trauma admission.

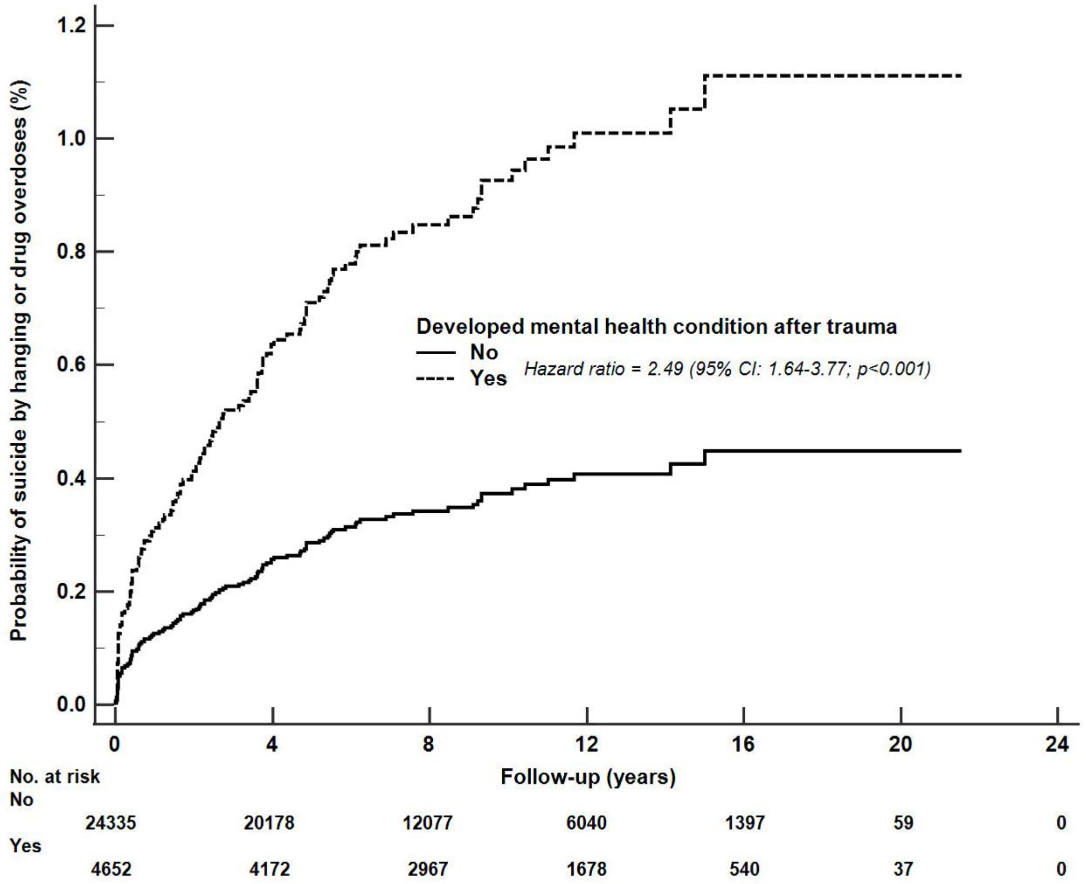

© 2025 Yaw LK et al. *JAMA Network Open.*

eFigure 2. Multivariable Cox regression showing the difference in risk of suicide by hanging or drug overdoses between those who developed a mental health condition and those without did not after their index trauma admission, after adjusted for age, Injury Severity Score (ISS), sex, and presence or absence of a mental health condition before the index trauma admission.

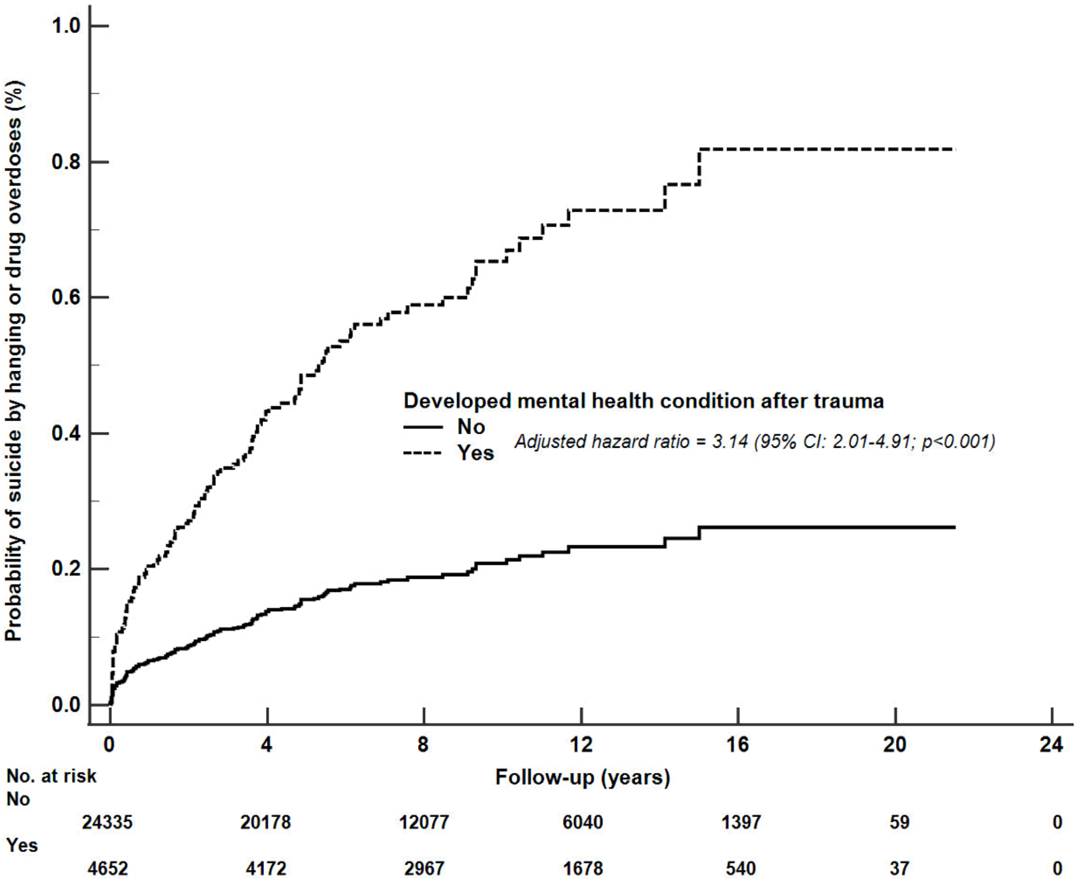

© 2025 Yaw LK et al. *JAMA Network Open*.

eTable. Predictors of developing a new mental health diagnosis after trauma among those who did not have any mental health conditions before their index trauma admissions (N=26,958).

**Multivariable logistic regression model including all those without any mental health conditions prior to index trauma admission (N=26,958)[a]**	**Odds ratio [OR] (95% confidence interval [CI])**	**P value**
Age *(OR per year increment)*	0.986 (0.982-0.989)	<0.001
Female *(vs male)*	0.917 (0.834-1.009)	0.08
Unemployed *(vs employed)* at the time of index trauma admission	2.867 (2.518-3.265)	<0.001
Indigenous	3.181 (2.813-3.598)	<0.001
Marital status at the time of index trauma admission: *(Married as the reference)*		
- Never married	1.270 (1.144-1.409)	<0.001
- Widowed	1.029 (0.854-1.240)	0.75
- Divorced	1.646 (1.343-2.016)	<0.001
- Separated	2.374 (1.906-2.956)	<0.001
- Unknown	1.329 (1.042-1.695)	0.02
Charlson comorbidity index *(OR per score increment)*	0.942 (0.893-0.993)	0.03
Socioeconomic status score[b] *(OR per score increment)*	0.999 (0.998-1.000)	<0.001
Traumatic brain injury as the principal injury at index trauma admission	1.095 (1.002-1.198)	0.04
Burns as the principal injury at index trauma admission	1.181 (0.948-1.470)	0.14
Penetrating injury	0.968 (0.844-1.110)	0.65

© 2025 Yaw LK et al. *JAMA Network Open.*

**Multivariable logistic regression model including only those who had data on smoking and alcohol use data but without any mental health conditions prior to index trauma admission (N=9820)**[c]	**Odds ratio [OR]**<br><br>**(95% confidence interval [CI])**	**P value**
Age *(OR per year increment)*	0.986 (0.982-0.990)	<0.001
Female *(vs male)*	0.951 (0.847-1.069)	0.40
Unemployed *(vs employed)* at the time of index trauma admission	2.779 (2.365-3.265)	<0.001
Indigenous	2.862 (2.460-3.331)	<0.001
Marital status at the time of index trauma admission:<br>*(Married as the reference)*<br>- Never married<br>- Widowed<br>- Divorced<br>- Separated<br>- Unknown	 <br><br>1.281 (1.126-1.457)<br>1.143 (0.912-1.434)<br>1.743 (1.368-2.222)<br>2.361 (1.787-3.118)<br>1.346 (0.960-1.886)	 <br><br><0.001<br>0.25<br><0.001<br><0.001<br>0.09
Charlson comorbidity index<br><br>*(OR per score increment)*	0.911 (0.853-0.974)	0.006
Socioeconomic status score[b]<br><br>*(OR per score increment)*	0.999 (0.998-0.999)	<0.001
Traumatic brain injury as the principal injury at index trauma admission	1.117 (1.000-1.248)	0.05
Burns as the principal injury at index trauma admission	1.317 (0.980-1.769)	0.07
Smoker *(vs non-smoker)*	1.397 (1.150-1.697)	<0.001
Alcohol user *(vs non-user)*	1.290 (1.076-1.546)	0.007
Penetrating injury	1.070 (0.927-1.234)	0.36

[a]Injury severity score, mechanism of injury (blunt vs penetrating), and neck injury as principal injured body region (all p values >0.25) were not significantly associated with having mental health condition after trauma and were removed from the model; the area under the receiver-operating-characteristic (ROC) curve of the model was 0.697 (95% CI 0.691-0.702). [b]A higher score indicates more advantaged based on socioeconomic factors, standardized to a distribution where the average equals 1,000 and the standard deviation is 100 (https://www.abs.gov.au/methodologies/socio-economic-indexes-areas-seifa-australia-methodology/2021). [c]The area under the receiver-operating-characteristic (ROC) curve of the model was 0.707 (95% CI 0.700-0.713).

© 2025 Yaw LK et al. *JAMA Network Open.*
