# Supplementary material for: Long-Term Outcomes and Determinants of New-Onset Mental Health Conditions After Trauma
Source: JAMA Netw Open. 2025 Mar 10;8(3):e250349. doi: 10.1001/jamanetworkopen.2025.0349 (PMC11894494; doi:10.1001/jamanetworkopen.2025.0349)
Supplement: Supplement 2. — Data Sharing Statement [file jamanetwopen-e250349-s002.pdf]

Data Sharing Statement

Yaw. Long-Term Outcomes and Determinants of New-Onset Mental Health Conditions After Trauma. *JAMA Netw Open*. Published March 10, 2025.
doi:10.1001/jamanetworkopen.2025.0349

Data
Data available: Yes
Data types: Deidentified participant data
How to access data: data will be made available after all sub-studies are completed. Please contact corresponding author
When available: With publication

Supporting Documents
Document types: None

Additional Information
Who can access the data: all reasonable requests
Types of analyses: for specified purposes
Mechanisms of data availability: after approval of a proposal
